# Extent of Non-Publication in Cohorts of Studies Approved by Research Ethics Committees or Included in Trial Registries

**DOI:** 10.1371/journal.pone.0114023

**Published:** 2014-12-23

**Authors:** Christine Schmucker, Lisa K. Schell, Susan Portalupi, Patrick Oeller, Laura Cabrera, Dirk Bassler, Guido Schwarzer, Roberta W. Scherer, Gerd Antes, Erik von Elm, Joerg J. Meerpohl, on behalf of the OPEN consortium

**Affiliations:** 1 German Cochrane Centre, Medical Center – University of Freiburg, Berliner Allee 29, 79110 Freiburg, Germany; 2 Institute of Medical Biometry and Statistics, Medical Center – University of Freiburg, Freiburg, Germany; 3 Department of Neonatology, University Hospital Zurich, Zurich, Switzerland; 4 Cochrane Switzerland, Institute of Social and Preventive Medicine (IUMSP), University Hospital Lausanne, Biopôle 2, Route de la Corniche 10, 1010 Lausanne, Switzerland; 5 US Cochrane Center, John Hopkins Bloomberg School of Public Health, Baltimore, Maryland, United States of America; UCL, United Kingdom

## Abstract

**Background:**

The synthesis of published research in systematic reviews is essential when providing evidence to inform clinical and health policy decision-making. However, the validity of systematic reviews is threatened if journal publications represent a biased selection of all studies that have been conducted (dissemination bias). To investigate the extent of dissemination bias we conducted a systematic review that determined the proportion of studies published as peer-reviewed journal articles and investigated factors associated with full publication in cohorts of studies (*i*) approved by research ethics committees (RECs) or (*ii*) included in trial registries.

**Methods and Findings:**

Four bibliographic databases were searched for methodological research projects (MRPs) without limitations for publication year, language or study location. The searches were supplemented by handsearching the references of included MRPs. We estimated the proportion of studies published using prediction intervals (PI) and a random effects meta-analysis. Pooled odds ratios (OR) were used to express associations between study characteristics and journal publication. Seventeen MRPs (23 publications) evaluated cohorts of studies approved by RECs; the proportion of published studies had a PI between 22% and 72% and the weighted pooled proportion when combining estimates would be 46.2% (95% CI 40.2%–52.4%, I^2^ = 94.4%). Twenty-two MRPs (22 publications) evaluated cohorts of studies included in trial registries; the PI of the proportion published ranged from 13% to 90% and the weighted pooled proportion would be 54.2% (95% CI 42.0%–65.9%, I^2^ = 98.9%). REC-approved studies with statistically significant results (compared with those without statistically significant results) were more likely to be published (pooled OR 2.8; 95% CI 2.2–3.5). Phase-III trials were also more likely to be published than phase II trials (pooled OR 2.0; 95% CI 1.6–2.5). The probability of publication within two years after study completion ranged from 7% to 30%.

**Conclusions:**

A substantial part of the studies approved by RECs or included in trial registries remains unpublished. Due to the large heterogeneity a prediction of the publication probability for a future study is very uncertain. Non-publication of research is not a random process, e.g., it is associated with the direction of study findings. Our findings suggest that the dissemination of research findings is biased.

## Introduction

The synthesis of published research in systematic reviews is essential when providing evidence to inform both clinical and health policy decision making. However, its validity is threatened if publications represent a biased selection of all the studies that have been conducted. Publication bias occurs when some types of results (e.g., those that are statistically significant) are reported more frequently or more quickly than others. [1]–[3] Increasingly, the term dissemination bias is used. It reflects that research reporting is not limited to journal publication alone but also comprises other forms of dissemination such as posting results in a trial registry. [4] Dissemination bias, similar to publication bias, results from favoured dissemination of research findings depending on their statistical significance and direction. It may lead to preferential prescribing of newer and more expensive treatments while underestimating the potential harm of drugs that have been in use for only a limited time. Clinical decisions may, therefore, be based on erroneous information. [5] It is obvious that these selection mechanisms violate the fundamental scientific and ethical imperative that findings from all research on humans should be available to advance knowledge. Furthermore, non-publication of studies implies considerable financial investment by funders without any return. Further down the road, indirect costs incurred due to non-publication of studies include those by health care providers, health insurances, and patients who all continue to pay for treatments that may not be the most effective ones or may even be harmful.

In response to these concerns, the OPEN Project (To Overcome failure to Publish nEgative fiNdings; www.open-project.eu) was developed with the goal of elucidating the scope of non-publication of studies through a series of systematic reviews and to develop recommendations. [4], [6]–[8] The OPEN Project was funded by the European Commission and conducted by an international working group of methodologists and other experts (see S1 Fig.). Besides evaluating the extent of non-publication of studies, OPEN examined current publication practices of key groups in the field of biomedical research (e.g., funding agencies, pharmaceutical industry, research ethics committees [RECs], trial registries, biomedical journals and regulatory agencies) through surveys and analysis of current policies and guidelines.

Because unpublished studies are hidden from view it is challenging to study dissemination bias. [9] One opportunity for such research is that in virtually all research settings REC approval is required before clinical studies can start. In addition, an increasing number of journals require prospective trial registration as a pre-condition for acceptance of manuscript reporting on studies. Further, any clinical study conducted under FDA regulations in the United States needs to be registered in clinicaltrials.gov (http://clinicaltrials.gov/ct2/manage-recs/fdaaa#WhichTrialsMustBeRegistered). Thus, study protocols submitted to RECs and study data accessible in trial registries are a resource to identify unpublished studies and evaluate the extent of non-publication of clinical research.

This systematic review investigated the extent to which studies approved by RECs or included in trial registries remained unpublished. To this effect, evidence from methodological research projects (MRPs) following such studies was evaluated and summarised. In addition, we assessed study characteristics that are potentially associated with publication (dissemination bias). The review was conducted according to a protocol published previously. [6]

## Methods

### Systematic literature search

We searched the databases Medline (Ovid), Embase (Ovid), The Cochrane Library and Web of Science from their inception until February 2012. An update search was performed in November 2013. The search strategy was based on combinations of medical subject headings (MeSH) and keywords and was not restricted to specific languages or years of publication. The search strategy used in Medline (Ovid) is presented in S2 Fig. Search strategies for other databases were adapted to meet the requirements of each database while keeping the search algorithm. The searches were supplemented by checking the bibliographies of any eligible articles for additional references. In addition, several experts in the field were contacted and asked to identify additional studies.

### Study selection and inclusion criteria

Titles and abstracts were reviewed using specific inclusion criteria (see below). All stages of study selection, data extraction and quality assessment were done independently by two reviewers (CS, LC, PO, LKS). Any disagreement during the selection, extraction, and assessment process was resolved by discussion and consensus.

We included MRPs which reported the proportion of studies published as journal publication after (*i*) REC approval or (*ii*) inclusion in trial registries. MRPs evaluating approved studies were included under the assumption that the majority of those studies were completed at the time of the search for peer-reviewed journal publications. In the case of multiple publications we extracted data from the MRP with the largest sample size and/or most comprehensive information while using cross-referencing.

### Outcomes

Our main outcomes were the overall proportion of studies published as journal articles and time to journal publication after study completion. Thereby, study completion was defined as the last day of follow-up of study participants. If the last day of follow-up was not given, time to publication was calculated based on the time reported in the MRP. To calculate the overall proportion of studies published, we set a minimum follow-up time of 24 months after study completion. In addition, we aimed to identify study characteristics that were associated with an increased likelihood of journal publication and time to publication. [6] We also collated information on costs or other resources which occurred by studies that were not published (if available). For the evaluation of study associations with publication a minimum follow-up time after study completion was not necessary. Outcomes were reported separately for both types of MRPs (RECs and trial registries).

### Data extraction and risk of bias assessment

Information on main characteristics of studies were abstracted for each MRP. [6] Internal and external validity of the identified MRPs was evaluated according to pre-defined criteria which were developed considering relevant literature investigating dissemination bias [10] and internal discussion. [6] Criteria for internal validity were: (*i*) follow-up time between study completion and search for journal publication, (*ii*) methodology used to identify journal publications, (*iii*) matching between study protocol or trial registry entry and retrieved journal publication and (*iv*) adjustment for confounders. External validity was judged based on the status of the study sample (i.e., whether the reported proportion of studies published was calculated based on a sample of completed and/or approved studies) and the sampling method used (i.e., whether a random or selected study sample was considered).

For each criterion an MRP's risk of bias was categorized as high, low or unclear.

### Statistical analysis/data synthesis

For both types of MRPs (RECs and trial registries) we separately estimated the proportion of studies published as journal articles using a random effects meta-analysis (DerSimonian-Laird method) based on logit-transformed proportions and their corresponding 95% confidence intervals (CI). Heterogeneity was assessed with the Chi^2^-test and calculation of the I^2^ statistic. [11] Given the substantial heterogeneity found we also decided to calculate prediction intervals (PI) - which were not pre-specified in the published protocol - using the method suggested by Higgins et al. [12] Pooled odds ratios (OR) were used to express associations between study characteristics and the likelihood of journal publication. Multivariate analyses of study characteristics were not feasible due to the small number of studies providing this information.

To address potential bias due to approved rather than completed studies, the status of the study sample (completed, on-going and/or approved) was evaluated within a sensitivity analysis.

Time to publication was analysed in two ways: (*i*) Mean or median time was used only if the proportion of studies published as peer-reviewed journal articles was larger than 50%. Some MRPs calculated time to publication from approval of studies to journal publication, others from study completion to journal publication. Due to these differences in definitions we refrained from pooling time-to-publication estimates. (*ii*) The proportion of studies published up to fixed time points (e.g., 6, 12, 18, 24, 36 months) was extracted from included MRP publications (e.g., from published Kaplan Meier curves). For each time point we performed a random-effects meta-analysis using logit-transformed proportions.

Statistical analyses were done with R using the *meta* package (http://cran.r-project.org/web/packages/meta/index.html).

## Results

### Results of literature search and selection process

**Figure 1:**
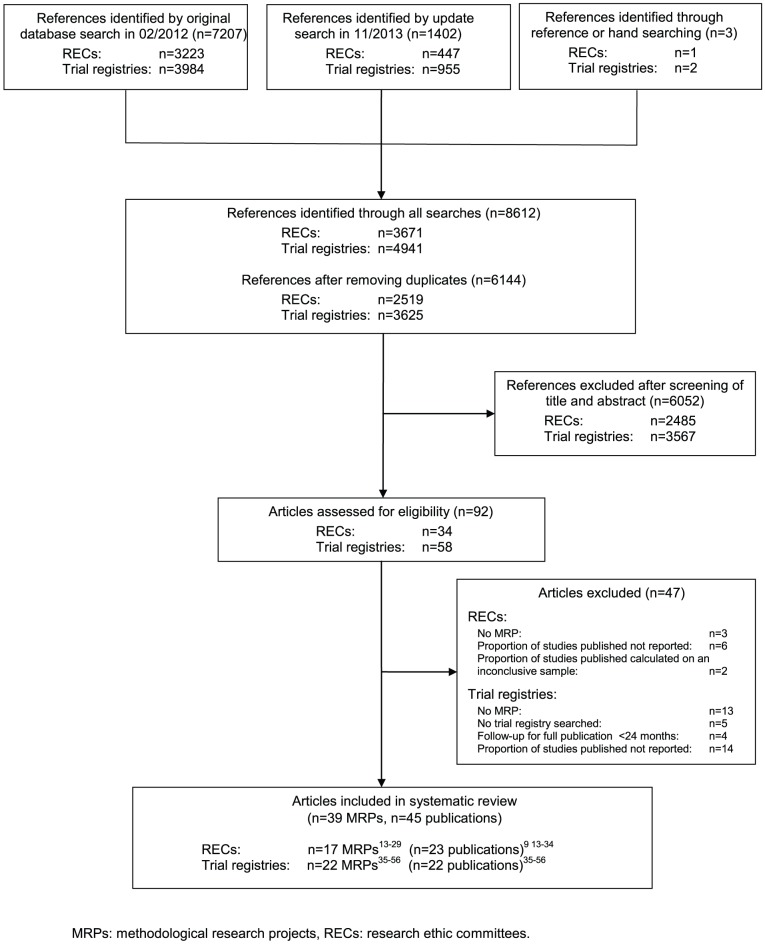
PRISMA statement flow diagram.

The searches identified 8612 references, including 2468 duplicates (Fig. 1). Among the 6144 potentially relevant references, 39 MRPs (45 publications) were eligible for the systematic review: 17 reported on MRPs following studies approved by RECs [13]–[29] (23 publications) [9], [13]–[34] and 22 on MRPs [35]–[56] following studies included in trial registries (22 publications).

### Characteristics of included MRPs

The main characteristics of included MRPs of both types are presented in Table 1 and Table 2.

**Table 1:** Main characteristics of 17 methodological research projects following studies after approval by a research ethics committee.

Reference	Medical field	Country of REC(s)	Included study sample	Methodology used to identify journal publications	Minimal time from approval/completion to search for journal publication (months)	No of selected studies	No of published studies (%)
**Blümle 2014 ** [13], [30], [31]	Not reported	Germany	All initiated human research studies approved by REC of the University of Freiburg (Germany) between 2000 and 2002 (studies of all designs)	Electronic database search, author contact (90% response rate)	104 (from approval)	807	419 (51.9)
**Chan 2004 ** [14]	Not reported	Denmark	RCTs approved by the Scientific-Ethical Committees for Copenhagen & Frederiksberg (Denmark) in 1994–1995 (RCTs)	Electronic database search, author contact (55% response rate)	>24 months* (from approval)	274	102 (37.2)
**Cooper 1997 ** [15]	Psychology	USA	Studies approved by the Department of Psychology Human Subjects Committee during the years 1986/87 and 1987/88 (study design not given)	Authors contact (100% response rate)	>24 months*(from approval)	159	41 (25.8)
**De Jong 2010 ** [16]	Epidemiology	The Netherlands	Random sample of approved protocols of epidemiological studies clinical trials between 1997 and 2006 (interventional+observational studies)	Electronic database search, author contact (100% response rate)	>24 months* (from approval)	80	23 (28.8)
**Decullier 2005 ** [17], [32]	Different medical specialties	France	Completed research protocols which had been approved in 1994 by a random sample of French committees (study design not given)	Author contact (100% response rate)	>24 months*	501	190 (37.9)
**Dickersin 1992 ** [18], [33]	Not reported	USA	Studies approved in 1980 or prior by 2 institutional review boards (observational+experimental studies)	Authors contact (100% response)	96 (from approval)	514	390 (75.9)
**Easterbrook 1992 ** [9], [19]	Different medical specialties	UK	Research protocols approved/completed by the Central Oxford REC between 1984 and 1987 (RCT+others)	Author contact (100% response)	29 (from completion)	285 (completed)/487 (approved)	138 (48.2)/209 (43.0)
**Hall 2007 ** [20]	Different medical specialties	Canada	All protocols submitted to the Capital District Health Authority Research Ethics Board for the period 1995–96 (RCTs+others)	Electronic database search, author contact (response rate not given)	>24 months* (from approval)	190	84 (44.2)
**Menzel 2007 ** [21]	Different medical specialties	Germany	Clinical trials approved in 1996 (study design not given)	Electronic database search	>24 months* (from approval)	99	71 (71.7)
**Olofsson 2000 ** [22]	Not reported	Sweden	All approved projects in 1992 (study design not given)	Electronic database search, author contact (response rate not given)	72 (from approval)	133	58 (43.6)
**Pich 2003 ** [23]	Different medical specialties	Spain	Clinical trials submitted in 1997 to REC (study design not given)	Author contact (response rate not given)	>24 months* from approval)	123	26 (21.1)
**Rodriguez 2009 ** [24]	Pediatrics	Argentina	Approved protocols between 01/2001 and 06/2006 (observational studies+others)	Author contact (response rate not given)	>24 months* (from approval)	125	40 (32)
**Stern 1997 ** [25]	Not reported	Australia	A cohort of studies submitted between 09/1979 and 12/1988 to a hospital ethics committee (RCT+others)	Author contact (100% response)	42 (from completion)	321	189 (58.9)
**Sune 2013 ** [26], [34]	Different medical specialties	Spain	Completed or prematurely terminated drug-related clinical trials approved by a general hospital ethics committee between 1997 and 2004 (controlled and uncontrolled studies)	Database search	>24 months* (from completion)	785	380 (48.4)
**Turer 2007 ** [27]	Different medical specialties	USA	All prospective, multicenter clinical trials of treatment approved in 1998 (observational studies excluded)	Electronic database search, author contact (response rate not given)	>24 months* (from approval)	197	101 (51.3)
**Von Elm 2008 ** [28]	Different medical specialties	Switzerland	Clinical studies of drug interventions submitted and completed to REC from 1988 to 1998 (RCTs)	Electronic database search, author contact (response rate not given)	>24 months* (from completion)	451	233 (51.7)
**Wise 1996 ** [29]	General Medicine	UK	First 100 consecutive protocols submitted and completed after establishment of REC by pharmaceutical companies (study design not given)	Contacted pharmaceutical companies/investigators (100% response)	>24 months* (from completion)	68	30 (44.1)

*No definite follow-up time predictable, but more than 24 months follow-up from study approval or completion to search for full publication fulfilled.

RCT: randomised controlled trial, REC: research ethic committee.

**Table 2:** Main characteristics of 22 methodological research projects following studies included in trial registries.

Reference	Medical field	Trial registry	Included study sample	Methodology used to identify journal publications	Minimal time from registration/completion to search for journal publication (months)	No of selected studies	No of published studies (%)
**Bourgeois 2010 ** [35]	Drug trials/internal medicine and psychiatry	clinicaltrials.gov	Safety and efficacy trials conducted and completed between 2000 and 2006 (observational studies+others)	Electronic database and trial registry search, author contact	39 (from completion)	546	362 (66.3)
**Chahal 2012 ** [46]	Orthopaedic sports medicine	clinicaltrials.gov	All RCTs related to sports medicine closed and completed by 06/2009 (RCTs)	Electronic database and trial registry search	33 (from completion)	34	20 (58.8)
**Gandhi 2011 ** [36]	Orthopaedic traumatology	clinicaltrials.gov	Interventional trials related to orthopaedic trauma registered and completed up to 07/2009 (study design not given)	Electronic database and trial registry search	>24 months* (from completion)	37	21 (56.8)
**Gopal 2012 ** [37]	Different medical specialties	clinicaltrials.gov	Completed interventional studies that may be subject to FDA regulation, 1 year prior to required results reporting (10/2006 to 09/2007) and during 2 years after required reporting (10/2007 to 09/2009) (study design not given)	Electronic database and trial registry search	25 (from completion)	818	185 (22.6)
**Guo 2013 ** [47]	Gynecology	clinicaltrials.gov	Interventional trials on endometriosis completed by 01/2012 (study design not given)	Electronic database search	24 month follow-up not fulfilled, but included to derive associations with full publications	27	5 (14.3)
**Hurley 2012 ** [38]	Cystic fibrosis (pneumology)	clinicaltrials.gov	Completed interventional trials between 01/1998 and 12/2010 (study design not given)	Electronic database search, author contact	24 month follow-up not fulfilled, but included to derive associations with full publications	142	75 (52.8)
**Huser 2012 ** [48]	Not reported	clinicaltrials.gov	Random sample of trials completed between 09/2004 and 12/2008 with no linked publication (study design not given)	Electronic database search, author contact	36 (from completion)	50	22 (44.0)
**Huser 2013 ** [49]	Not reported	clinicaltrials.gov	Completed interventional trials between 01/2006 and 12/2009 (Phase-2 studies)	Electronic database and trial registry search	38 (from completion)	8907	2477 (27.8)
**Jones 2013 ** [50]	Not reported	clinicaltrials.gov	Large (>500 patients) studies that were prospectively registered and closed prior to 01/2009 (RCTs)	Electronic database and trial registry search, author contact	46 (from completion or closed trials) (minimal follow-up)	513	381 (74.3)
**Khan 2012 ** [51]	Rheumatology	clinicaltrials.gov	Trials of drug therapy for rheumatoid arthritis of phase 2 or higher and completed between 2002–2003 or between 2006–2007 (RCTs)	Electronic database and trial registry search	Follow-up not given, but included to derive associations with full publications/or sensitivity analysis	62	42 (67.7)
**Liu 2010 ** [39]	Different medical specialties	clinicaltrials.gov and 10 WHO registries	Completed Chinese interventional trials (observational studies+others)	Electronic database and trial registry search, author contact	24 month follow-up not fulfilled, but included to derive associations with full publications	443	156 (35.9)
**Prenner 2011 ** [40]	Ophthalmology	clinicaltrials.gov	Completed interventional trials (study design not given)	Electronic database search	>24 months* (from completion)	64	35 (54.7)
**Ramsey 2008 ** [41]	Oncology	clinicaltrials.gov	Interventional trials designated as either completed or terminated in 09/2007 (RCTs+others)	Electronic database and trial registry search	24 month follow-up not fulfilled, but included to derive associations with full publications	2028	357 (17.6)
**Ross 2009 ** [42]	Different medical specialties	clinicaltrials.gov	Clinical trials registered after 01/2000 and updated as having been completed by 01/2006 excluding phase I trials (observational studies+others)	Electronic database and trial registry search, author contact	>24 months* (from completion)	677	311 (46.0)
**Ross 2012 ** [43]	Different medical specialties	clinicaltrials.gov	Interventional trials funded by NIH registered after 09/2005 and updated as having been completed by 01/2009 (study design not given)	Electronic database and trial registry search	26 (from completion)	635	432 (68.0)
**Shamliyan 2012 ** [53]	Urology	clinicaltrials.gov	Completed or discontinued trials of drug therapies or nonsurgical treatments for women with urinary incontinence (interventional+observational studies)	Electronic database and trial registry search	>24 months* (from completion)	112	26 (23.2)
**Shamliyan 2012 ** [52]	Pediatrics	clinicaltrials.gov	Random sample of completed pediatric trials (interventional +observational studies)	Not reported	24 month follow-up not fulfilled, but included to derive associations with full publications	758	218 (28.8)
**Smith 2012 ** [54]	Orthopaedics	clinicaltrials.gov	Closed RCTs on arthroplasty with an estimated completion date up to 07/2009 (RCTs)	Electronic database and trial registry search	24 month follow-up not fulfilled, but included to derive associations with full publications or sensitivity analysis	101	23 (22.8)
**Tfelt-Hansen 2011 ** [44]	Neurology	GSK trial registry	RCTs (double-blind) concerning the use of naratriptan in migraine (RCTs)	Search in GSK registry	Follow-up not given, but included to derive associations with full publications or sensitivity analysis	17	11 (64.7)
**Thorn 2013 ** [55]	Not reported	ISRCTN register	RCTs planning an economic evaluation with an anticipated end before 01/2008 (RCTs)	Electronic database and trial registry search, authors contact	>24 months* (from completion)	100	70 (70.0)
**Vawdrey 2013 ** [56]	Not reported	clinicaltrials.gov	Clinical trials of electronic health records completed by 2009 (study design not given)	Electronic database and trial registry search, author contact	>24 months* (from completion)	62	47 (75.8)
**Wildt 2011 ** [45]	Gastroenterology, Hepatology	clinicaltrials.gov	RCTs (phase III) on adult patients with gastrointestinal diseases initiated or completed during 1998 and 2008 (RCTs)	Electronic database search	>24 months* (from initiation or completion)	105	66 (62.9)

*No definite follow-up time predictable, but more than 24 months follow-up from study completion to search for full publication fulfilled.

FDA: US Food and Drug Administration, GSK: Glaxo Smith Kline, ISRCTN: International Standard Randomised Controlled Trial Number, NIH: National Institutes of Health, RCT: randomised controlled trial, WHO: World Health Organisation.

#### MRPs following studies after REC approval

Of the 17 MRPs that followed studies approved by RECs, four focused on specific medical fields: psychology, [15] epidemiology, [16] paediatrics [24] and general medicine [29]. Eight [17], [19]–[21], [23], [26]–[28] included studies from different fields and five [13], [14], [18], [22], [25] did not provide any information. Two MRPs [14] [28] included solely randomised controlled trials and 15 allowed for a wide range of interventional and observational study designs. The RECs in charge of study approval were based in Germany, [13], [21] USA, [15], [18], [27] The Netherlands, [16] Denmark, [14] France, [17] United Kingdom, [19], [29] Canada, [20] Sweden, [22] Spain, [23], [26] Argentina, [24] Australia [25] and Switzerland. [28]

#### MRPs following studies after inclusion in trial registries

Of the 22 MRPs following studies included in trial registries, 12 focused on specific medical fields: orthopaedics, [36], [46] pneumology, [38] ophthalmology, [40] oncology, [41] neurology, [44] gynecology, [47] rheumatology, [51] urology, [53] pediatrics, [52] orthopedics [54] and gastroenterology/hepatology [45]. One MRP included drug trials in internal medicine and psychiatry [35] and another was restricted to Chinese trials dealing with different medical specialties. [39] Three MRPs did not restrict their field of research [37], [42], [43] and five did not provide any information. [48]–[50], [55], [56] Seven MRPs included randomised controlled trials. [44]–[46], [50], [51], [56] The remaining MRPs either did not specify included study designs or included a wide range of designs ranging from observational studies to controlled clinical trials. Twenty [35]–[43], [45]–[54], [56] of the 22 MRPs searched www.clinicaltrials.gov. Besides clinicaltrials.gov one MRP also searched 10 WHO registries for Chinese trials, [39] two other MRPs searched the GlaxoSmithKline (GSK) trial registry (United Kingdom) [44] and the ISRCTN register, [55] respectively.

### Risk of bias

Results of the methodological quality assessment are presented in Table 3 and Table 4, respectively.

**Table 3:** Risk of bias table for MRPs following studies after approval by a REC.

	Internal validity				External validity	
	follow-up time between study completion and search for journal publication	methodology used to identify journal publications	matching between REC protocol and journal publication	adjustment for confounders	research status of REC protocol (e.g., approved or completed trial)	sampling method (e.g., all trials, random sample)
Blümle 2014 [13], [30], [31]	?	+	+	-	-	-
Chan 2004 [14]	?	+	?	-	-	?
Cooper 1997 [15]	?	+	NA	-	-	?
De Jong 2010 [16]	?	+	?	-	-	+
Decullier 2005 [17], [32]	+	+	NA	-	+	+
Dickersin 1992 [18] [33]	?	+	NA	-	-	+
Easterbrook 1992 [19] [9] *	+	+	NA	-	+	+
Hall 2007 [20]	?	+	-	-	-	+
Menzel 2007 [21]	?	+	-	-	-	?
Olofsson 2000 [22]	?	+	?	-	-	+
Pich 2003 [23]	?	?	NA	-	-	?
Rodriguez 2009 [24]	?	?	NA	-	-	?
Stern 1997 [25]	+	+	NA	-	-	+
Sune 2013 [26] [34]	+	+	+	-	+	+
Turer 2007 [27]	?	+	?	-	-	+
Von Elm 2008 [28]	+	+	+	-	+	?
Wise 1996 [29]	+	+	NA	-	+	+

*Easterbrook 1992 reported publication rates for completed and approved studies separately. We just refer to the completed sample in this review. NA: Not applicable.

**+** means low risk of bias; **-** means high risk of bias;**?** means unclear risk of bias.

**Follow-up time:**>24 months after study completion: **+**. <24 months follow-up after study completion: **-**. MRPs which judged their follow- up rather on approved than completed studies. Although these MRPs fulfilled the 24 month follow-up criteria, we judged them to have an unclear risk of bias:**?**.

**Methodology used to identify journal publication:** electronic search and author contact: **+**. only author contact (with response rate of ≥80%): **+**. only author contact (with response rate <80%): **-**; only database search (in 1 database): **-**. only database search (in>1 database): **+**. methodology not given:**?**.

**Adjustment for confounders:** if an analysis for factors associated with journal publication was carried out: **+**. if no analysis was carried out: **-**.

**Matching criteria:** if ≥2 matching criteria given: **+**. if <2 matching criteria given: **-**. matching criteria not given in MRP:**?**. if only author contact was used to identify journal publication: **NA** (not applicable).

**Research status:** completed protocols: **+**. approved protocols: **-**.

**Sampling method:** all trials, random sample or consecutive trials: **+**. if only investigator responded to questionnaire for this sample: **-**. sampling method not given (e.g., without the word “all”):**?**.

**Table 4:** Risk of bias table for MRPs following studies included in trial registries.

	Internal validity				External validity	
	follow-up time between study completion and search for journal publication	methodology used to identify journal publications	matching between registry entry and journal publication	adjustment for confounders	research status of registry entry (e.g., completed or ongoing trial)	sampling method (e.g., all trials, random sample)
Bourgeois 2010 [35]	+	+	?	-	+	+
Chahal 2012 [46]	+	+	+	-	+	+
Gandhi 2011 [36]	+	+	+	-	+	+
Guo 2013 [47]	-	-	-	-	+	+
Gopal 2012 [37]	+	+	?	-	+	+
Hurley 2012 [38]	-	+	?	-	+	+
Huser 2012 [48]	+	+	?	-	+	+
Huser 2013 [49]	+	+	?	-	+	+
Jones 2013 [50] *	+	+	+	-	+	+
Khan 2012 [51]	?	+	+	-	+	+
Liu 2010 [39]	-	+	+	-	+	+
Prenner 2011 [40]	+	-	?	-	+	+
Ramsey 2008 [41] *	-	+	?	-	+	+
Ross 2009 [42]	+	+	+	-	+	+
Ross 2012 [43]	+	+	+	-	+	+
Shamliyan 2012a [53] *	+	+	?	-	+	+
Shamliyan 2012b [52]	-		?	-	+	+
Smith 2012 [54]	-	+	+	-	+	+
Tfelt-Hansen 2011 [44] **	?	-	?	-	?	+
Thorn 2013 [55]	+	+	?	-	+	+
Vawdrey 2013 [56]	+	+	?	-	+	+
Wildt 2011 [45]	+		?	-	-	

*The research status of Shamliyan 2012a refers to completed and terminated trials.

**The MRP of Tfelt-Hansen 2011 is based on the GKS registry only.

**+** means low risk of bias; **-** means high risk of bias;**?** means unclear risk of bias.

**Follow-up time:**>24 months: **+**. <24 months: **-**. follow-up time not given/or could not be estimated:**?**.

**Methodology used to identify journal publication:** electronic search and author contact and/or search in trial registry: **+**. only author contact (with a response rate of ≥80%): **+**. only author contact (with a response rate <80%): **-**. only search in trial registry or only 1 database: **-**. methodology not given:**?**.

**Adjustment for confounders:** if an analysis for factors associated with the journal publication was carried out: **+**. if no analyses were carried out: **-**.

**Matching criteria:** if ≥2 matching criteria given: **+**. if <2 matching criteria given: **-**. matching criteria not given in MRP:**?**.

**Research status:** completed registry entries: **+**. completed and initiated mixed: **-**. not mentioned:**?**.

**Sampling method:** all trials, random sample or consecutive trials: **+**. if only investigator responded to questionnaire for this sample: **-**. sampling method not given (e.g., without the word “all”):**?**.

#### MRPs following studies after REC approval

All of the included MRPs fulfilled the 24-month follow-up criterion. However, twelve MRPs based their follow-up time on studies which were approved but not necessarily completed. [13]–[16], [18], [20]–[25], [27] Although these MRPs fulfilled the 24-month follow-up criterion, we judged them to have an unclear risk of bias. The methodology used to identify journal publications was adequate in all but two MRPs. [23], [24] Three MRPs performed adequate matching between protocol and retrieved journal publications. [13], [26], [28] However, in most MRPs this criterion was not applicable because identification of journal publications relied solely on author contacts. None of the MRP publications reported on adjustment for confounding factors when calculating proportions of published studies in specific subgroups or calculating measures of association between likelihood of publication and subgroup characteristics.

#### MRPs following studies after inclusion in trial registries

Fourteen MRPs following studies included in trial registries had a follow-up time between study completion and search for full publication of 24 months or more. [35]–[37], [40], [42], [43], [45], [46], [48]–[50], [53], [55], [56] All but one [45] of these MRPs included cohorts of completed studies. The publication status was verified by searching adequate electronic databases and/or contacting the lead investigators in all but two MRPs. [44], [52] Thirteen MRPs [35], [37], [38], [40], [41], [44], [45], [48], [49], [52], [53], [55], [56] did not comment on matching criteria between registry entry and retrieved journal publication; whereas all but one [47] remaining MRPs performed adequate matching. Similar to MRPs following studies after REC approval, adjustment for confounders was not considered in any of the MRPs.

### Proportion of studies published

**Figure 2:**
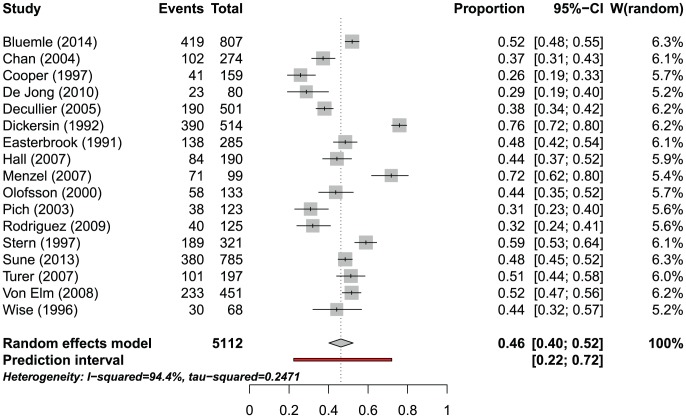
Weighted proportion of published studies for 17 MRPs following studies after REC approval.

After REC approval, the proportion of studies published ranged from 26% to 76% in 17 MRPs with a follow-up of 24 months or more, including 5112 studies (Fig. 2, Table 5). The prediction interval was 22% to 72%; the heterogeneity among individual estimates was substantial (I^2^ = 94.4%, p<0.0001). If one combined the individual estimates even so the pooled estimate would be 46.2% (95% CI 40.2–52.4).

**Table 5:** Pooled proportions of published studies based on methodological research projects.

MRP category	No of MRPs	Weighted proportion of studies published (95% CI)	Heterogeneity test: I^2^ (p value Chi^2^ test)	Prediction Interval
RECs [9], [13]–[18], [20]–[29]	17	46.2% (40.2–52.4)	94.4% (<0.0001)	22% – 72%
Trial registries [35]–[37], [40], [42], [43], [45], [46], [48]–[50], [53], [55], [56]	14	54.2% (42.0–65.9)	98.9% (<0.0001)	13%–90%

CI: Confidence interval, MRP: Methodological research project, No: Number, REC: Research ethics committee.

**Figure 3:**
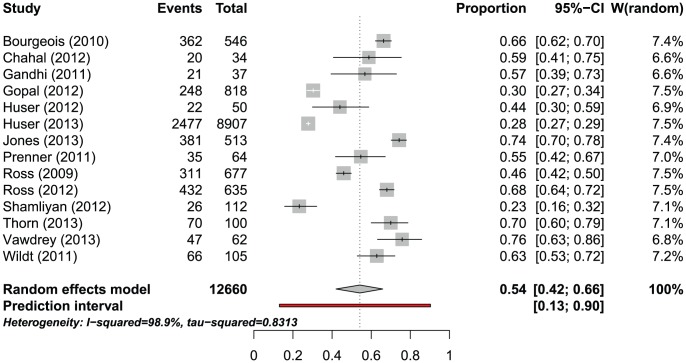
Weighted proportion of published studies for 14 MRPs following studies after trial registration.

After trial registration, the proportion of studies published ranged from 23% to 76% in 14 MRPs with a follow-up of 24 months or more, including 12660 studies (Fig. 3, Table 5). The prediction interval was 13% to 90%; again the heterogeneity among individual estimates was substantial (I^2^ = 98.9%, p<0.0001). If one combined the individual estimates even so the pooled estimate would be 54.2% (95% CI 42.0–65.9).

In a sensitivity analysis we excluded those MRPs that were based on a cohort of approved [13]–[16], [18], [20]–[25], [27] or initiated [45] studies. In the resulting sample of completed studies a pooled proportion of studies published would be similar: 46.3% (95% CI 41.0–51.6; I^2^ = 81.1%, p<0.0001; based on five MRPs following studies after REC approval) [17], [19], [26], [28], [29] and 53.5% (95% CI 40.9–65.7; I^2^ = 98.9%, p = 0.0003; based on 13 MRPs following studies after inclusion in trial registries) [35]–[37], [40], [42], [43], [46], [48]–[50], [53], [55], [56], respectively. The sensitivity analysis of MRPs which only included randomised controlled trials would yield a pooled proportion of published studies of 44.5% (95% CI 31.0–58.8; I^2^ = 92.9%, p = 0.0002; based on two MRPs following studies after REC approval) [14], [28] and 60.3% (95% CI 45.4–73.6; I^2^ = 92.5%, p<0.001; based on seven MRPs following studies after trial registration), respectively. [44]–[46], [50], [51], [54], [55] It should be noted that three of these MRPs had insufficient follow-up time for searching full publications [44], [51], [54] or included on-going studies. [45]

### Factors associated with publication

Table 6 summarizes factors associated with journal publication of studies. Four MRPs following studies approved by RECs compared studies with statistically significant results (p<0.05) and studies with non-significant results. [9], [18], [25], [26] The pooled OR for publication of studies with statistically significant results (vs. non-significant) was 2.8 (95% CI 2.2–3.5). Also studies with positive results defined as experimentally better or clinically relevant had higher (but statistically not significant) odds of journal publication than studies with negative results (pooled OR 3.1; 95% CI 0.9–11.0; two MRPs). [25], [32]

**Table 6:** Factors associated with journal publication.

	No of MRPs	Pooled OR (95% CI)	Heterogeneity test: I^2^ (p value of Chi^2^ test)
**Significant vs non-significant results**			
RECs [9], [18], [25], [26]	4	2.8 (2.2–3.5)	0.0% (0.79)
Trial registries	nr		
**Experimentally better vs not better results**			
RECs [25], [32]	2	3.1 (0.9–11.0)	76.9% (0.04)
Trial registries [56]	1	11.7 (2.8–48.5)	- (−)
**Phase III vs phase II studies**			
RECs [26]	1	1.5 (1.0–2.0)	- (−)
Trial registries [37], [38], [40]–[43], [47], [50], [52], [53]	10	2.0 (1.6–2.5)	22.0% (0.24)
**RCTs vs observational studies**			
RECs [20], [30]	2	2.0 (1.3–3.3)	0.0% (0.69)
Trial registries [39], [42], [43]	3	1.2 (1.0–1.5)	0.0% (0.78)
**Basic vs human research**			
RECs [24], [30]	2	1.1 (0.6–2.1)	49.0% (0.16)
Trial registries	nr		
**Multicentre vs single centre studies**			
RECs [18], [28], [30], [34]	4	1.5 (1.0–2.4)	49.0% (0.12)
Trial registries	nr		
**National vs international**			
RECs [30]	1	1.1 (0.6–1.8)	- (−)
Trial registries [40]	1	1.3 (0.5–38)	- (−)
**Funding vs no funding**			
RECs [18]	1	3.2 (2.0–5.2)	- (−)
Trial registries	nr		
**Government vs industry funding**			
RECs [26]	1	1.2 (0.8–1.9)	- (−)
Trial registries [37], [39], [41], [42], [46], [50], [52], [53]	8	2.2 (1.7–2.9)	43.8% (0.09)
**Sample size>vs sample size <than median**			
RECs	nr		
Trial registries [42]	1	1.2 (0.8–1.6)	- (−)

Nr: Not reported, MRP: Methodological research project, No: Number, OR: Odds ratio, REC: Research ethics committee.

Two of the MRPs that followed studies after REC approval [20], [30] and three of the MRPs that followed studies after registration [39], [42], [43] investigated the association of different study designs with publication (i.e., randomised controlled trials versus observational studies). In both types of MRPs, randomised controlled trials had a greater odds of publication than observational studies (OR 2.0; 95% CI 1.3–3.3 and 1.2; 95% CI 1.0–1.5, respectively). A post-hoc analysis including MRPs that followed studies after trial registration revealed that phase-III trials were more likely to be published than phase-II trials (pooled OR 2.0; 95% CI 1.6–2.5). [37], [38], [40]–[43], [47], [50], [52], [53]

In MRPs that followed studies after REC approval, multicentre studies were more likely to be published than single centre studies (pooled OR 1.5; 95% CI 1.0–2.4; four MRPs). [18], [28], [30], [34] We also found that research funded by governments was more frequently published than research funded by the industry (pooled OR 2.2; 95% CI 1.7–2.9; eight MRPs following studies after trial registration). [37], [39], [41], [42], [46], [50], [52], [53] But no difference in the probability of publication between basic and human research was identified (pooled OR 1.1; 95% CI 0.6–2.1; two MRPs). [24], [30] There were also no significant differences for national versus international studies (OR 1.3; 95% CI 0.5–3.8) in one MRP following studies after inclusion in trial registries [40] and for studies with sample sizes larger (versus smaller) than the cohort's median sample size (OR 1.2; 95% CI 0.8–1.6) in another such MRP. [42] Other potential factors associated with journal publications could not be derived from the included MRPs. In addition, none of the MRPs reported on costs or use of other resources due to studies that were not published.

### Factors associated with time to publication

The time to full publication of studies being published in peer-reviewed journals was reported in two MRPs following studies after REC approval: [17], [26] One MRP [17] reported a statistically significant (p<0.001) association of the direction of results with mean time to publication of 62.4 months (95% CI 57.6–67.2) for positive (confirmatory) results compared with 78 months (95% CI 69.6–86.4) for studies with inconclusive results and 82.2 months (95% CI 70.8–94.8) for negative (invalidating) results. The second MRP [26] confirmed that the time to publication is significantly associated with the direction of the results. Median time to full publication was 25 months in studies with positive outcomes and 38.5 months in those with negative results.

### Probability of publication over time

**Figure 4:**
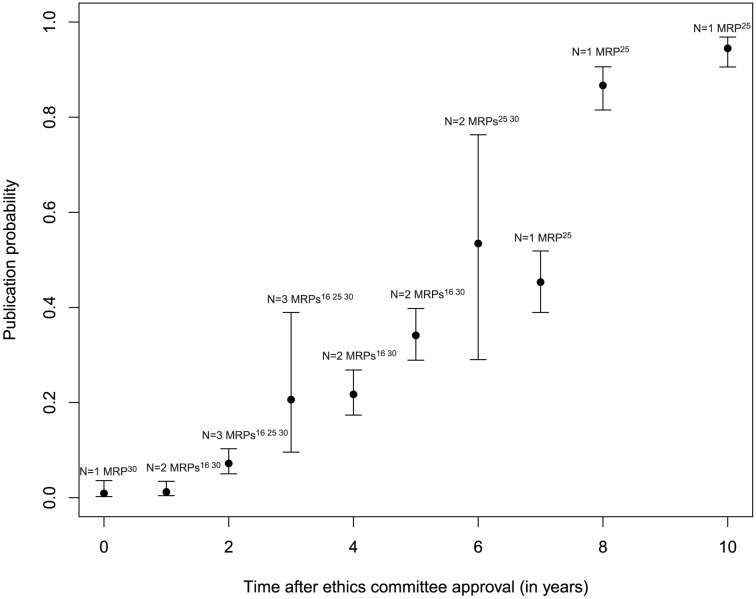
Time to publication after ethics committee approval.

**Figure 5:**
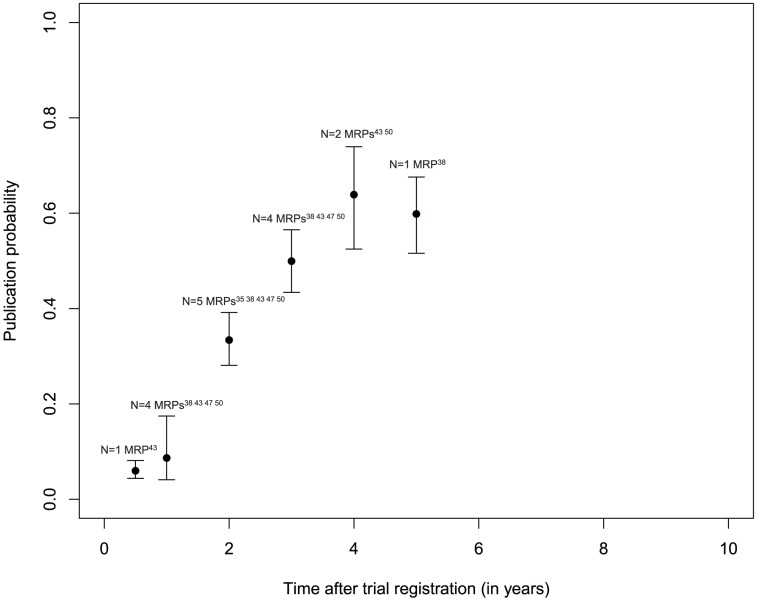
Time to publication after trial registration.

Three MRPs following studies approved by RECs provided information on the time course of publication (Fig. 4): [16], [25], [30] after two years the publication probability was approximately 7%, [16], [25], [30] after three years 20%, [16], [25], [30] after five years 30%, [16], [30] and after six years 55%. [25], [30] Estimates of publication probability after trial registration were available from five MRPs (Fig. 5): [35], [38], [43], [47], [50] After two years the publication probability reached approximately 30%, [35], [38], [43], [47], [50] after three years 50%, [38], [43], [47], [50] and after five years approximately 60%. [38] Because of the low number of included MRPs with data on follow-up, these estimates have to be interpreted cautiously.

## Discussion

### Principal findings

Overall, only about half of clinical and preclinical studies approved by RECs or included in trial registries are published as full journal articles; however estimates vary largely resulting in wide prediction intervals. For randomised controlled trials a pooled overall proportion of studies published would be somewhat larger (60.3%; 95% CI 45.4–73.6). Accordingly, prediction of the probability of publication for a future study is very uncertain. We also found evidence for dissemination bias in that studies with statistically significant results were more likely to be published than those without (pooled OR 2.8; 95% CI 2.2–3.5). This association is consistent with the finding that studies with positive results defined as experimentally better or clinically relevant were more likely to be published than studies with negative results though not reaching statistical significance (pooled OR 3.1; 95% CI 0.9–11.0). In addition, phase-III trials – which might be more successful than early-phase trials – were more likely to be published than phase-II trials (pooled OR 2.0; 95% CI 1.6–2.5). Also, randomised controlled trials which are considered as the “gold standard” design for a clinical study are published more often than observational studies (pooled OR 2.0; 95% CI 1,3–3,3). The reason for this finding could be that medical journals prefer to publish randomised controlled trials. But there may also be a tendency by study authors not to not write up results of observational studies, in particular when they are negative.

### Strengths and weaknesses of this review

The findings of our systematic review are based on a thorough and comprehensive literature search for the available evidence on dissemination bias. We considered two types of MRPs which tracked studies from time of inception, thus including 39 individual MRPs evaluating more than 20,000 studies. For both types, the evidence on dissemination bias was consistent suggesting that publications over the last 20 years are an incomplete and biased subset of research findings. We conducted our systematic review following a pre-specified protocol thus preventing that any substantial post-hoc changes remain undisclosed. [6] Because a registry for methodological reviews is not yet available, this protocol was not prospectively registered, but previously published in an open-access journal. [6]

Our systematic review may have some limitations. We identified a large number of MRPs but associations between study characteristics and journal publication had not been reported in most of these publications. Therefore, not all pre-specified subgroup analyses stated in the protocol could be performed. For example, it was not possible to collate enough data on sex and rank of lead investigator or language of publication to investigate these factors in association with non-publication. We could not determine with certainty whether the MRP authors carried out additional analyses that ultimately were not reported (as authors were not contacted personally), thus selecting outcomes for publication. Furthermore, the aggregated data for publication probability over time refer to less than five studies at most time points. Accordingly, publication probabilities at given time points have to be interpreted cautiously. The reported estimates can only give a rough picture of the publication course after REC approval or trial registration. No standard methodology is available to assess study quality of the types of research projects we considered for our review. Therefore, we devised a tool to assess internal and external validity of the identified evidence. A sensitivity analysis for MRPs with high risk of bias was planned initially [6] but not performed due to the overall low quality of the identified MRPs. When we calculated the overall proportion of studies published as journal articles we only included studies with an arbitrarily defined minimum follow-up time of 24 months after study completion. Therefore, the proportion of studies published may be underestimated in some MRPs because journal publication may have occurred later. We included MRPs which investigated approved or on-going rather than completed studies. A sensitivity analysis excluding those MRPs showed that the proportion of studies published was similar. In addition, limited data on potential risk factors (e.g., follow-up time, language of included studies) made it impossible to further explore the large heterogeneity observed in our data. We also acknowledge shortcomings (like inaccurate estimation of heterogeneity) of random effects models meta-analysis - as carried out in our systematic review - with a small number of included studies.

### Comparison with other systematic reviews

In a Cochrane Methodology Review full publication of results initially presented in abstracts was examined combining data from 79 MRPs; the weighted mean full publication proportion was 44.5% (95% CI 43.9–45.1). [10] In this review, survival analyses combining aggregated data resulted in an estimated publication rate at nine years of 52.6% for all types of studies, 63.1% for randomised controlled trials and 49.3% for other types of study designs. In addition, the review showed a significant association of positive study results (defined as any statistically significant result) with full publication. Other factors associated with full publication were randomised trial study design and funded research. Despite the different criteria for inclusion of MRPs (REC approval/trial registration versus meeting presentation) their findings were consistent with our results.

The extent of dissemination bias in different types of research projects was also investigated by Song et al. 2009. [57] The authors identified 12 MRPs that followed up research from inception (studies approved by RECs or registered by research funding bodies), four MRPs that included trials submitted to regulatory authorities, 28 MRPs that assessed the fate of studies presented as conference abstracts, and four MRPs that followed manuscripts submitted to journals. This review concluded that dissemination bias related to direction of study results mainly occurs before the presentation of findings at conferences and the submission of manuscripts to journals. [57] A recent systematic review of studies limited to randomised trials confirmed the existence of dissemination bias and outcome reporting bias, although meta-analysis was not conducted due to the differences between included studies. [58] In addition, a Cochrane Review concluded that trials with positive findings are published more often and more quickly than trials with negative findings. [59] Despite differences in types of study cohorts or MRPs included, all these reviews were consistent with our body of evidence in concluding that a study with positive findings is more likely to be published than a study with negative results. One might speculate that journals prefer publishing reports with positive rather than non-positive results or that investigators do not submit reports of studies with negative results.

### Implications for policy makers and further research

Overall, the scientific literature represents an incomplete subset of research findings. Due to the large heterogeneity, prediction of the probability of publication for a single study is very uncertain. Our findings clearly confirm that (non-)publication is not a random process and the likelihood of publication is associated with the direction of study findings. When results are not published or are published selectively based on the direction or the strength of the findings, healthcare professionals and consumers of healthcare cannot base their decisions on the full body of current evidence. This ignorance can lead to the use of ineffective or harmful interventions and to waste of scarce health-care resources. For example, when unpublished studies were included in a meta-analysis, the antidepressant reboxetine was shown to have more adverse effects but no better efficacy than placebo for treatment of major depression – a different finding from that when only published studies were included. [60]

The inability to make evidence-informed decisions impacts the healthcare system at various levels: First, dissemination bias is at odds with the ethical responsibility towards patients to use all research to advance medical knowledge and improve their care. Second, if treatment effects are overestimated this may result in patients receiving treatments that may be more harmful or less efficacious than previously believed. Finally, non-publication of studies results is deleterious because a considerable part of the funds available for research are spent without return. Additional costs include those incurred by health care systems and individual patients who continue to pay for treatments that may not be as effective or efficient as commonly thought. Although the full extent of financial impact of non-publication of studies is currently unknown, the waste of funds is likely to be high. [61], [62]

The creation of clinical trial registers and the prospective publication of detailed study protocols with explicit outcome descriptions and analysis plans should help to combat dissemination bias. The recent AllTrials campaign has proposed that “all trials past and present should be registered, and the full methods and the results reported” (http://www.alltrials.net/). In addition, researchers should be encouraged and supported to present their studies at conferences and to proceed until full publication.

Nevertheless, dissemination bias exists and is currently invalidating findings in systematic reviews and meta-analyses when only published studies are considered. There is no excuse for study results to go unpublished and there is a huge public health benefit from obtaining a complete picture of what has been found in all studies to-date.

## Supporting Information

S1 Fig **The OPEN Consortium.**
(DOCX)

S2 Fig **Search Strategy for OvidSP MEDLINE.**
(DOCX)

S1 PRISMA Checklist **PRISMA checklist.**
(DOC)
