# Supplementary material for: Extent of Non-Publication in Cohorts of Studies Approved by Research Ethics Committees or Included in Trial Registries
Source: PLoS One. 2014 Dec 23;9(12):e114023. doi: 10.1371/journal.pone.0114023 (PMC4275183; doi:10.1371/journal.pone.0114023)
Supplement: S1 Fig — The OPEN Consortium. (DOCX) [file pone.0114023.s001.docx]

**Figure S1. The OPEN Consortium.**

**OPEN Consortium**

| Contributor | Participating Institution |
| --- | --- |
| Antes, Gerd | German Cochrane Centre, Institute of Medical Biometry and Medical Informatics University Medical Center, Freiburg, Germany |
| Bassler, Dirk | Center for Pediatric Clinical Studies, University Medical Center Tuebingen, Germany |
| Bertele, Vittorio | Department of Epidemiology, Mario Negri Institute for Pharmacological Research, Italy |
| Bonfill, Xavier | The Clinical Epidemiology & Public Health Department at the Hospital de la Santa Creu i Sant Pau, Spain |
| Bouesseau, Marie-Charlotte | World Health Organization, Geneva, Switzerland |
| Boutron, Isabelle | INSERM U738 research unit, Paris Descartes University, Paris, France |
| Gallus, Silvano | Department of Epidemiology, Mario Negri Institute for Pharmacological Research, Italy |
| Garattini, Silvio | Department of Epidemiology, Mario Negri Institute for Pharmacological Research, Italy |
| Ghassan, Karam | World Health Organization, Geneva, Switzerland |
| La Vecchia, Carlo | Department of Epidemiology, Mario Negri Institute for Pharmacological Research, Italy |
| Lang, Britta | German Cochrane Centre, Institute of Medical Biometry and Medical Informatics University Medical Center, Freiburg, Germany |
| Littmann, Jasper | CELLS (Centre for Ethics and Law in Life Sciences), Hannover Medical Scholl, Hannover, Germany |
| Kleijnen, Jos | Kleijnen Systematic Reviews Ltd., York, United Kingdom |
| Kulig, Michael | Federal Joint Committee, Berlin, Germany |
| Malicki, Mario | University of Split School of Medicine, Split, Croatia |
| Marusic, Ana | University of Split School of Medicine, Split, Croatia |
| Meerpohl, Joerg* | German Cochrane Centre, Institute of Medical Biometry and Medical Informatics University Medical Center, Freiburg, Germany |
| Mueller, Katharina Felicitas | Center for Pediatric Clinical Studies, University Medical Center Tuebingen, Germany |
| Pardo, Hector | The Clinical Epidemiology & Public Health Department at the Hospital de la Santa Creu i Sant Pau, Spain |
| Perleth, Matthias | Federal Joint Committee, Berlin, Germany |
| Ravaud, Philippe | INSERM U738 research unit, Paris Descartes University, Paris, France |
| Reis, Andreas | World Health Organization, Geneva, Switzerland |
| Schmucker, Christine | German Cochrane Centre, Institute of Medical Biometry and Medical Informatics University Medical Center, Freiburg, Germany |
| Schwarzer, Guido | German Cochrane Centre, Institute of Medical Biometry and Medical Informatics University Medical Center, Freiburg, Germany |
| Strech, Daniel | CELLS (Centre for Ethics and Law in Life Sciences), Hannover Medical Scholl, Hannover, Germany |
| Trinquart, Ludovic | INSERM U738 research unit, Paris Descartes University, Paris, France |
| Urrútia, Gerard | The Clinical Epidemiology & Public Health Department at the Hospital de la Santa Creu i Sant Pau, Spain |
| von Elm, Erik | German Cochrane Centre, Institute of Medical Biometry and Medical Informatics University Medical Center, Freiburg, Germany Cochrane Switzerland, IUMSP, University Hospital Lausanne, Lausanne, Switzerland |
| Wager, Elizabeth | Sideview, Princes Risborough, United Kingdom |
| Wieland, Alexandra | Federal Joint Committee, Berlin, Germany |
| Wolff, Robert | Kleijnen Systematic Reviews Ltd., York, United Kingdom |

* Leader of the OPEN Consortium: Dr. Joerg J Meerpohl, email: meerpohl@cochrane.de
