# Supplementary material for: Extent of Non-Publication in Cohorts of Studies Approved by Research Ethics Committees or Included in Trial Registries
Source: PLoS One. 2014 Dec 23;9(12):e114023. doi: 10.1371/journal.pone.0114023 (PMC4275183; doi:10.1371/journal.pone.0114023)
Supplement: S2 Fig — Search Strategy for OvidSP MEDLINE. (DOCX) [file pone.0114023.s002.docx]

**Figure S2. Search Strategy for OvidSP MEDLINE.**

| *Ethics committes* | |
| --- | --- |
| 1 | exp Publishing/sn |
| 2 | *publishing/ |
| 3 | publication bias/ |
| 4 | selection bias/ |
| 5 | exp manuscripts as topic/ |
| 6 | ((data or finding? or information or evidence or study or studies or trial? or paper? or article? or report* or literature or work or manuscript? or abstract* or result?) adj6 (unpublish* or un-publish* or unreport* or un-report* or nonpublish* or non-publish* or nonpublicat* or non-publicat* or (publication? adj3 rate?) or "not publish*")).ti,ab. |
| 7 | (underreport* or under-report* or selective report* or selective publish* or selective publicat* or (final* adj2 (report* or publish* or publicat* or manuscript? or paper? or article?)) or (full? adj2 (report* or publish* or publicat* or manuscript? or paper? or article?)) or (subsequent* adj2 (report* or article? or paper? or publi* or manuscript?)) or (sub-sequent* adj2 (report? or article? or paper? or publi* or manuscript?)) or (complete* adj2 (report* or article? or paper? or publish* or publicat* or manuscript?))).ti,ab. |
| 8 | (bias* adj3 (publish* or publicat*)).ti,ab. |
| 9 | or/1-8 |
| 10 | exp Ethics Committees/ |
| 11 | exp Ethical Review/ |
| 12 | clinical protocols/ |
| 13 | ((institution* adj3 review* adj3 board?) or IRB? or (ethic? adj3 protocol?) or (ethic? adj3 committee?) or ((clinical adj3 protocol?) and ethic*) or ((study adj3 protocol?) and ethic*) or (ethic* adj3 (review* or approv*))).ti,ab. |
| 14 | or/10-13 |
| 15 | 9 and 14 |
| 16 | exp animals/ not humans/ |
| 17 | 15 not 16 |
| 18 | remove duplicates from 17 |
| *Trial registries* | |
| 1 | exp Publishing/sn |
| 2 | *publishing/ |
| 3 | publication bias/ |
| 4 | selection bias/ |
| 5 | exp manuscripts as topic/ |
| 6 | ((data or finding? or information or evidence or study or studies or trial? or paper? or article? or report* or literature or work or manuscript? or abstract* or result?) adj6 (unpublish* or un-publish* or unreport* or un-report* or nonpublish* or non-publish* or nonpublicat* or non-publicat* or (publication? adj3 rate?) or "not publish*")).ti,ab. |
| 7 | (underreport* or under-report* or selective report* or selective publish* or selective publicat* or (final* adj2 (report* or publish* or publicat* or manuscript? or paper? or article?)) or (full? adj2 (report* or publish* or publicat* or manuscript? or paper? or article?)) or (subsequent* adj2 (report* or article? or paper? or publi* or manuscript?)) or (sub-sequent* adj2 (report? or article? or paper? or publi* or manuscript?)) or (complete* adj2 (report* or article? or paper? or publish* or publicat* or manuscript?))).ti,ab. |
| 8 | (bias* adj3 (publish* or publicat*)).ti,ab. |
| 9 | or/1-8 |
| 10 | registries/ |
| 11 | (registry or registries or register?).ti. |
| 12 | (("ClinicalTrials.gov" adj6 regist*) or (Current Controlled Trials adj6 regist*)).ab. |
| 13 | or/10-12 |
| 14 | 9 and 13 |
| 15 | exp animals/ not humans/ |
| 16 | 14 not 15 |
| 17 | remove duplicates from 16 |
